# Supplementary material for: Discovery of novel glycerolated quinazolinones from Streptomyces sp. MBT27
Source: J Ind Microbiol Biotechnol. 2019 Feb 7;46(3):483–92. doi: 10.1007/s10295-019-02140-2 (PMC6403205; doi:10.1007/s10295-019-02140-2)
Supplement: Supplementary file 1 — Supplementary material 1 (PDF 859 kb) [file 10295_2019_2140_MOESM1_ESM.pdf]

Discovery of Novel Glycerolated Quinazolinones from *Streptomyces* sp. MBT27

Nataliia Machushynets [1], Changsheng Wu [1,3,] *, Somayah Sameer Elsayed [1], Thomas Hankemeier [2] and Gilles P. van Wezel [1,] *

[1] *Molecular Biotechnology, Institute of Biology, Leiden University, Sylviusweg 72, 2333 BE, The Netherlands*

[2] *Leiden Academic Centre for Drug Research (LACDR), Leiden University, Einsteinweg 55, 2333 CC, Leiden, The Netherlands*

[3] *State Key Laboratory of Microbial Technology, Institute of Microbial Technology, Shandong University, Qingdao 266237, P.R. China*

* Author for correspondence. Tel: +31 71 5274310; email: g.wezel@biology.leidenuniv.nl, or wcs0313@gmail.com

Figures list

Fig. S1 Effect of carbon source utilization on secondary metabolite profiles of *Streptomyces* sp. MBT27

Fig. S2 Changes in the secondary metabolite profiles of *Streptomyces* sp. MBT27 depending on the glycerol concentration

Fig. S3 ^{1}H NMR spectrum of *quinazolinone A* (**1**) in CD_3OD

Fig. S4 APT spectrum of *quinazolinone A* (**1**) in CD_3OD

Fig. S5 ^{1}H-^{1}H COSY spectrum of *quinazolinone A* (**1**) in CD_3OD

Fig. S6 HSQC spectrum of *quinazolinone A* (**1**) in CD_3OD

Fig. S7 HMBC spectrum of *quinazolinone A* (**1**) in CD_3OD

Fig. S8 ^{1}H-^{1}H NOSEY spectrum of *quinazolinone A* (**1**) in CD_3OD

Fig. S9 HRMS spectrum of *quinazolinone A* (**1**)

Fig. S10 UV spectrum of *quinazolinone A* (**1**)

Fig. S11 IR spectrum of *quinazolinone A* (**1**).

Fig. S12 ^{1}H NMR spectrum of *quinazolinone B* (**2**) in CD_3OD

Fig. S13 APT spectrum of *quinazolinone B* (**2**) in CD_3OD

Fig. S14 ^{1}H-^{1}H COSY spectrum of *quinazolinone B* (**2**) in CD_3OD

Fig. S15 HSQC spectrum of *quinazolinone B* (**2**) in CD_3OD

Fig. S16 HMBC spectrum of *quinazolinone B* (**2**) in CD_3OD

Fig. S17 ^{1}H-^{1}H NOSEY spectrum of *quinazolinone B*(**2**) in CD_3OD.

Fig. S18 HRMS spectrum of *quinazolinone B* (**2**)

Fig. S19 UV spectrum of *quinazolinone B* (**2**)

Fig. S20 IR spectrum of *quinazolinone B* (**2**)

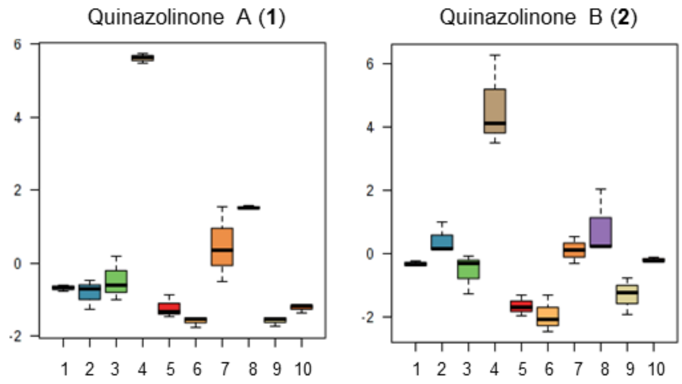

Fig. S1 Effect of carbon source utilization on the secondary metabolite profiles of *Streptomyces* sp. MBT27. Box plots show the relative intensities of quinazolinones A (**1**) and B (**2**) in cultures grown in MM with different carbon sources, namely: 1. mannitol 1% + glycerol 1%; 2. GlcNAc 1%; 3. glycerol 1%; 4. glycerol 2%; 5. mannitol 1%; 6. mannitol 2%; 7. glucose 1%; 8. glucose 2%; 9. arabinose 1%; 10. fructose 1%. Note the spectacular increase in production when the glycerol concentration is doubled.

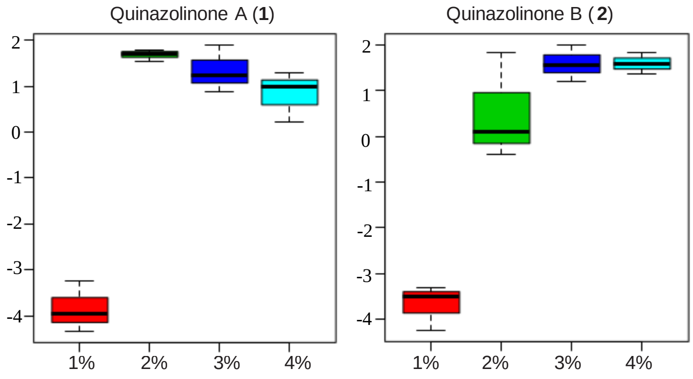

Fig. S2 Changes in the secondary metabolite profiles of *Streptomyces* sp. MBT27 depending on the glycerol concentration. Box plots show the relative intensities of quinazolinones A (**1**) and B (**2**) in the cultures with added 1%, 2%, 3% or 4% (w/v) glycerol.

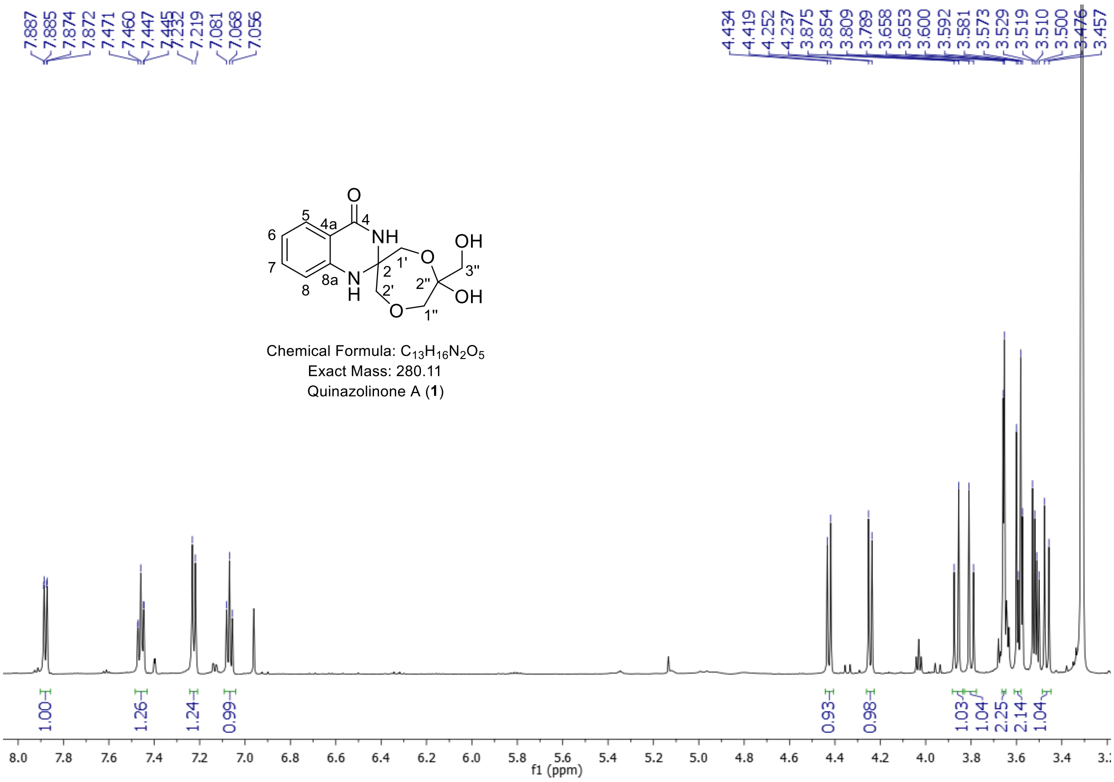

Fig. S3 ^{1}H NMR spectrum of *quinazolinone A* (**1**) in CD_3OD

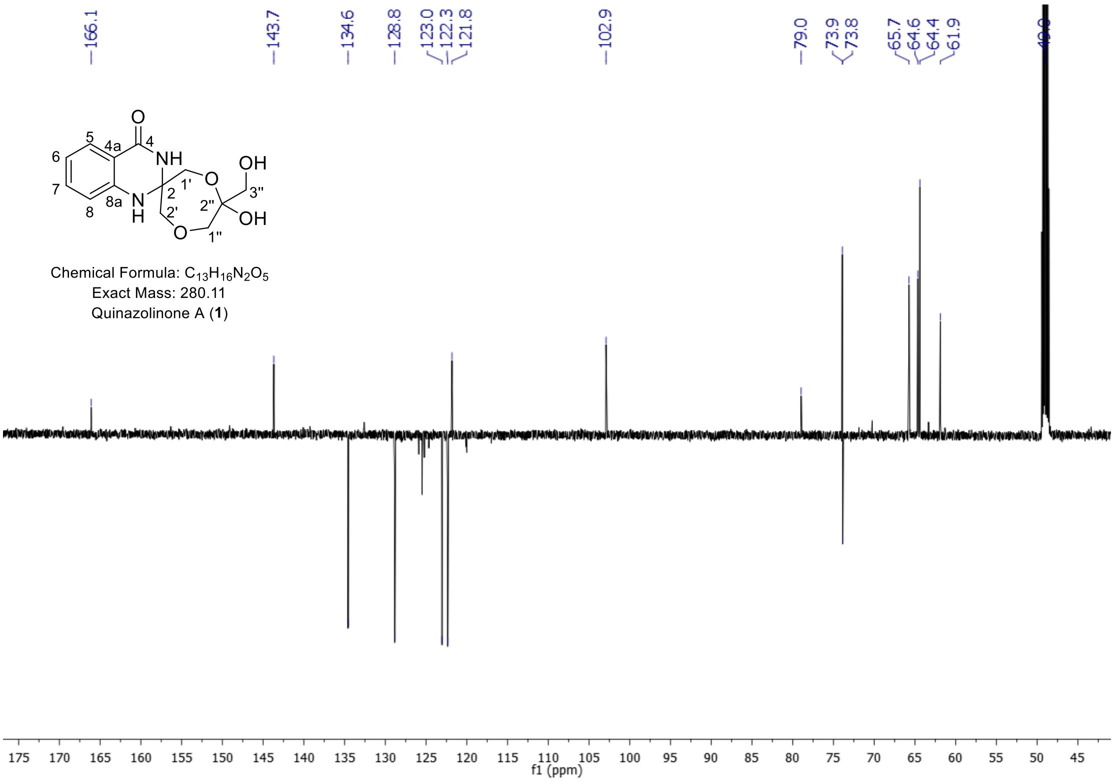

Fig. S4 APT spectrum of *quinazolinone A* (**1**) in CD_3OD

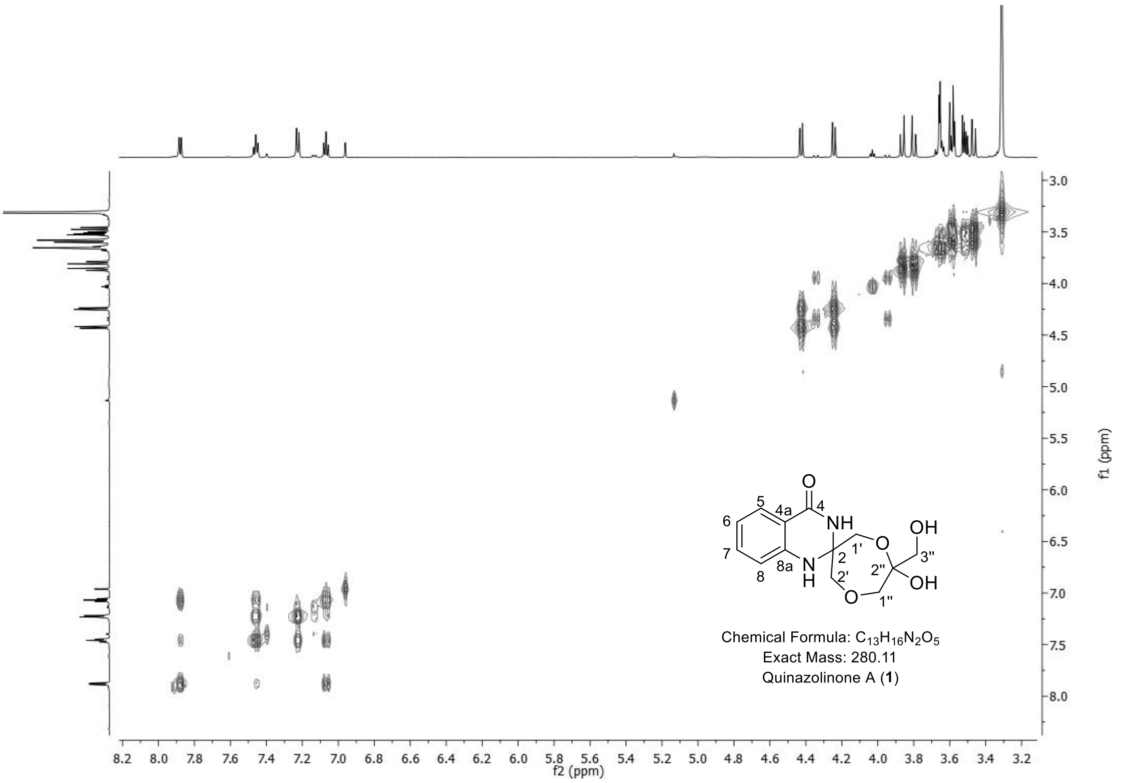

Fig. S5 ^{1}H-^{1}H COSY spectrum of *quinazolinone A* (**1**) in CD_3OD

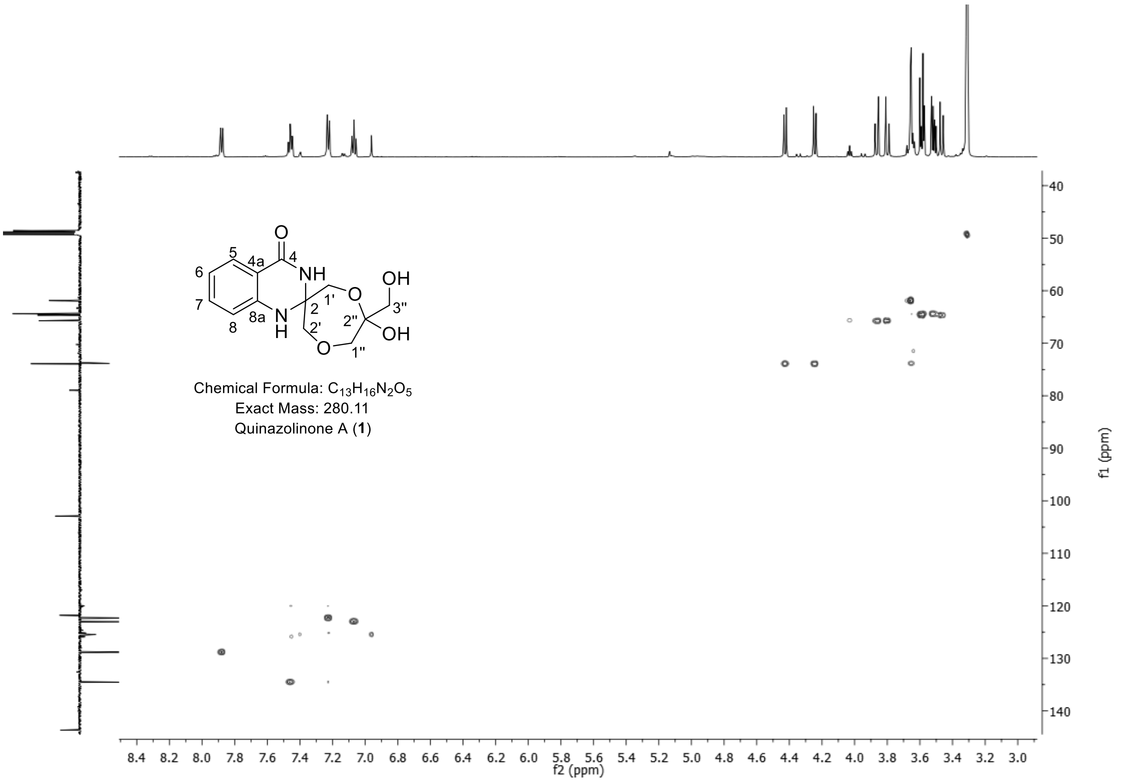

Fig. S6 HSQC spectrum of *quinazolinone A* (**1**) in CD_3OD

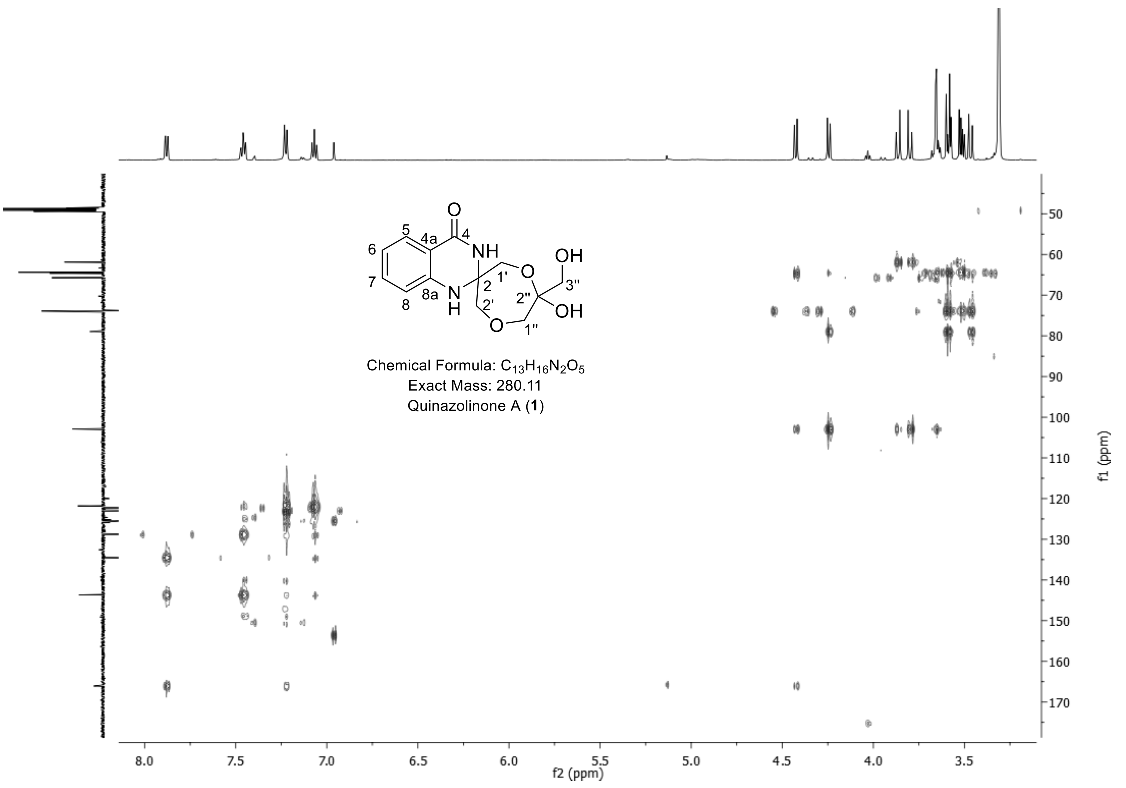

Fig. S7 HMBC spectrum of *quinazolinone A* (**1**) in CD_3OD

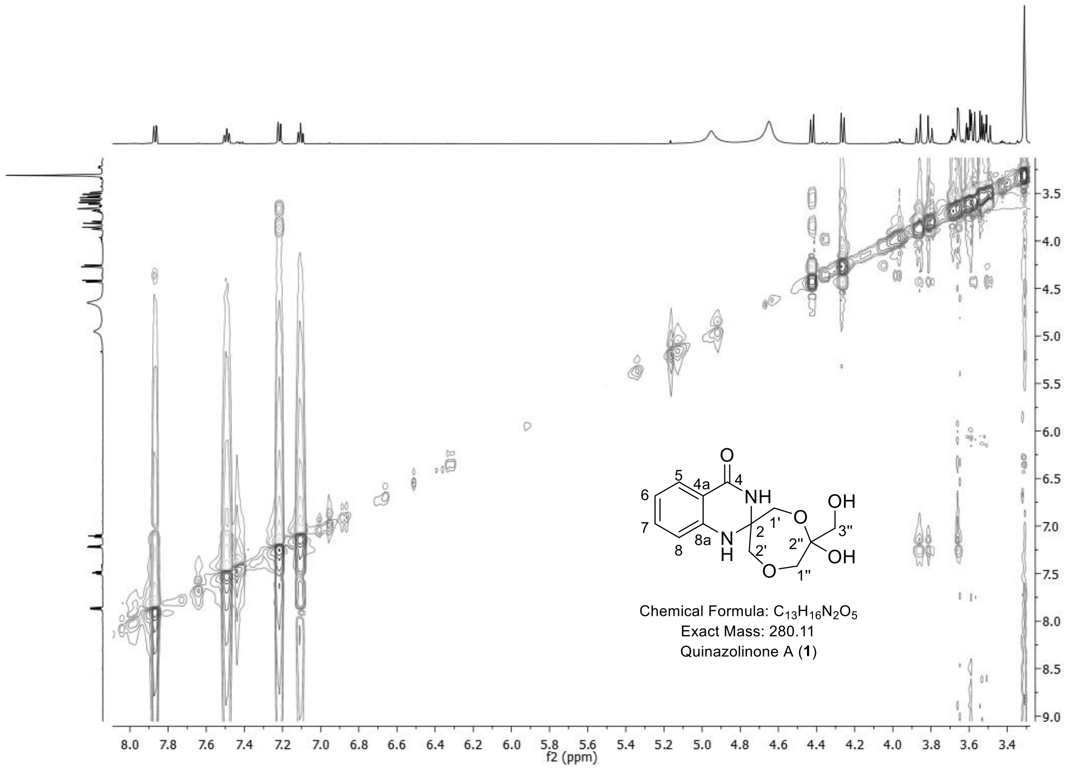

Fig. S8 ^{1}H-^{1}H NOSEY spectrum of *quinazolinone A* (**1**) in CD_3OD

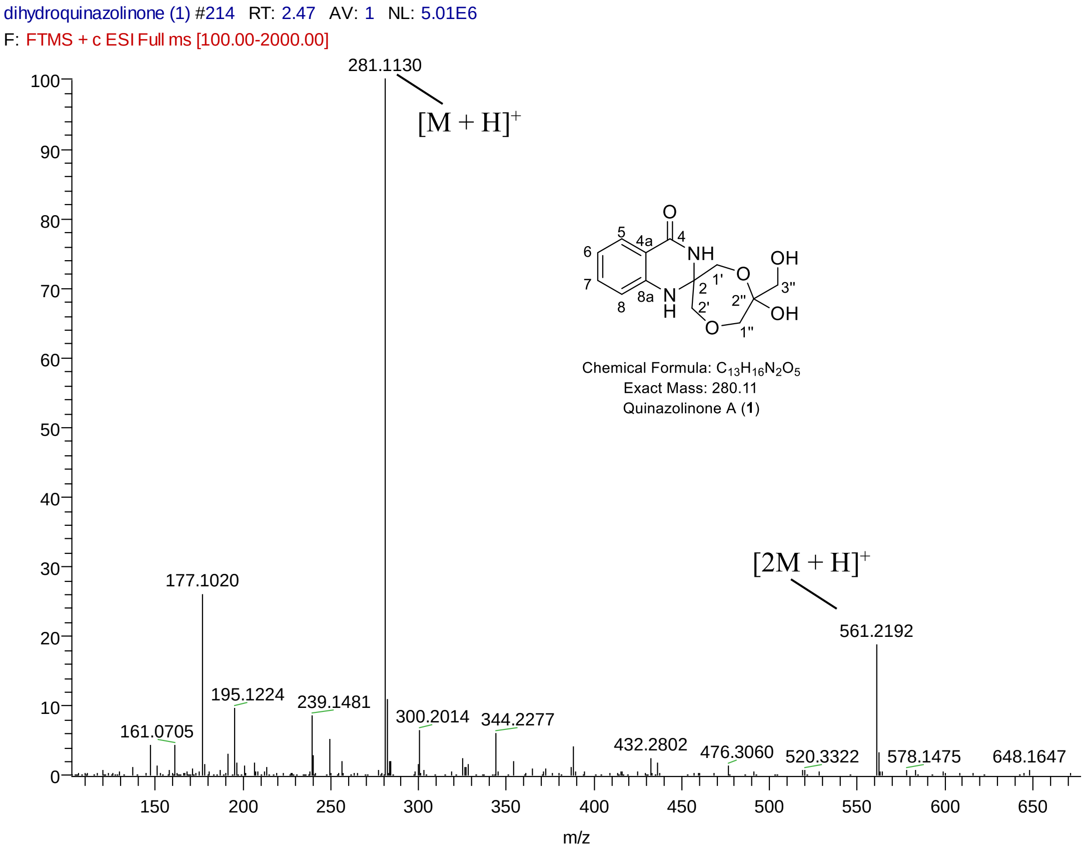

Fig. S9 HRMS spectrum of *quinazolinone A* (**1**)

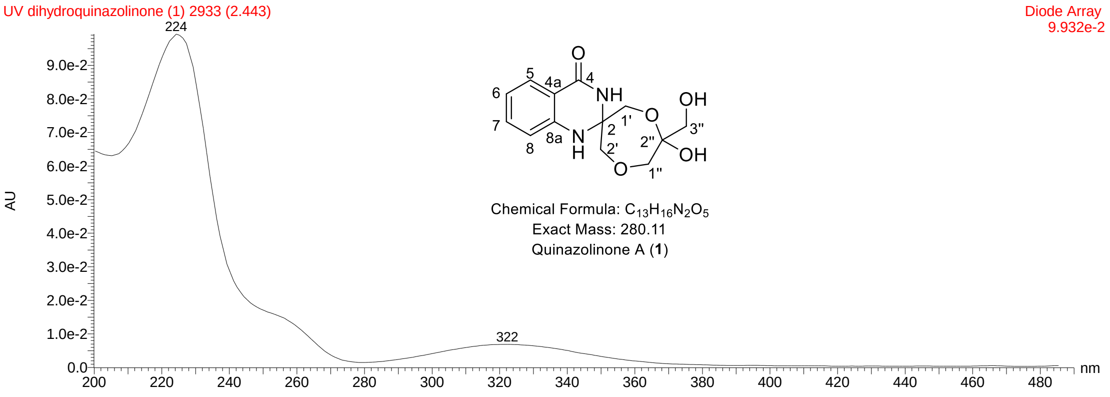

Fig. S10 UV spectrum of *quinazolinone A* (**1**)

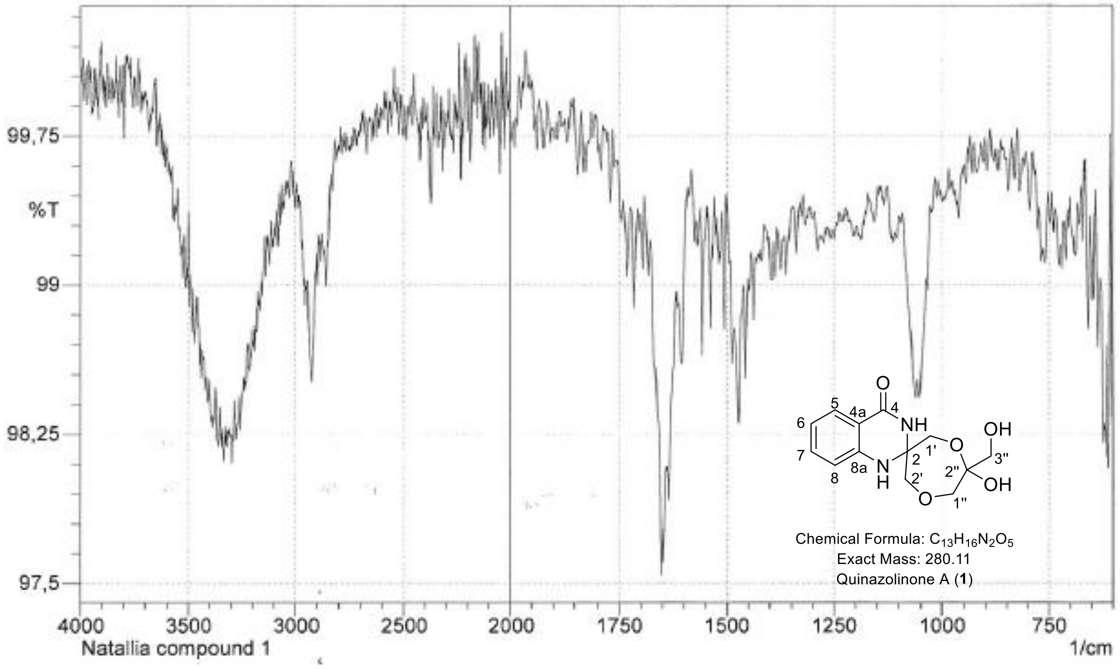

Fig. S11 IR spectrum of *quinazolinone A* (**1**)

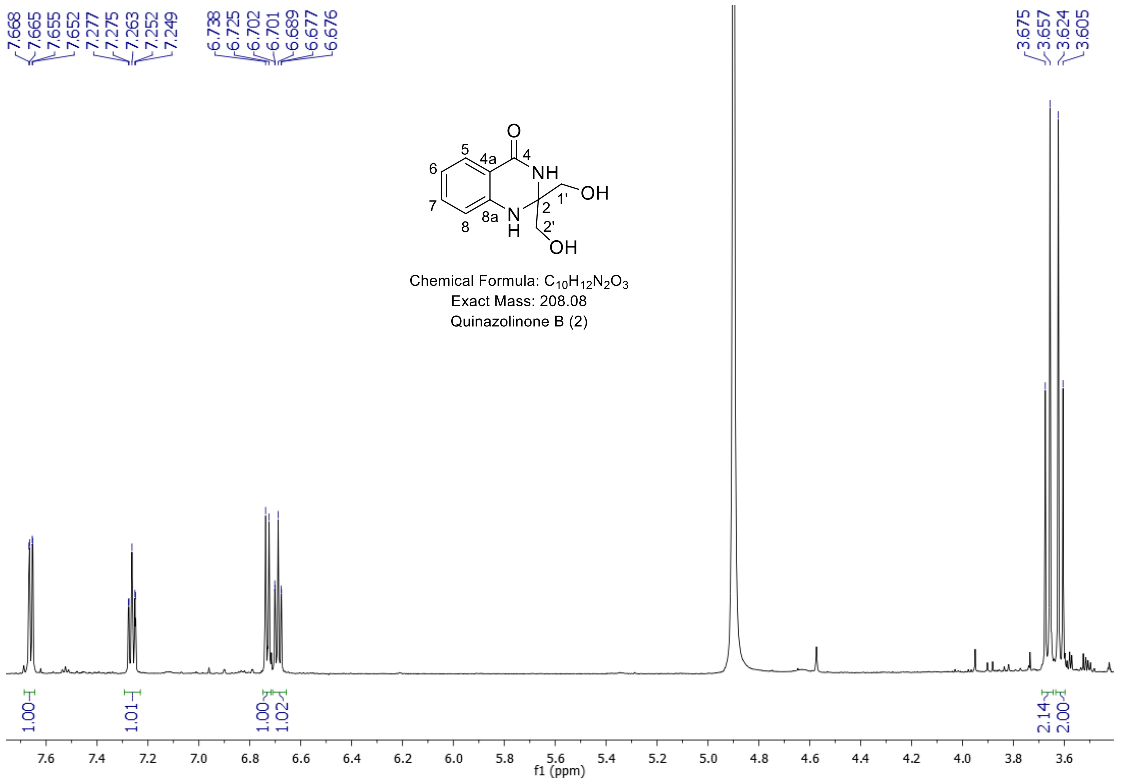

Fig. S12 ^{1}H NMR spectrum of *quinazolinone B* (**2**) in CD_3OD

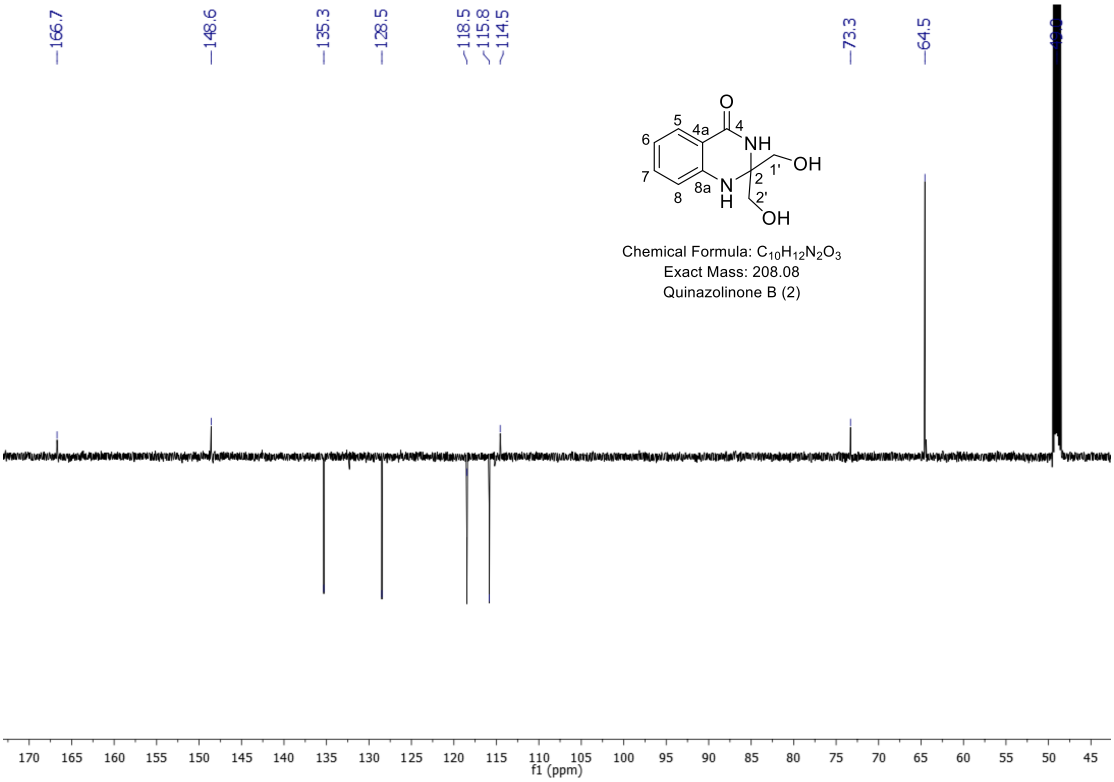

Fig. S13 APT spectrum of *quinazolinone B* (**2**) in CD_3OD

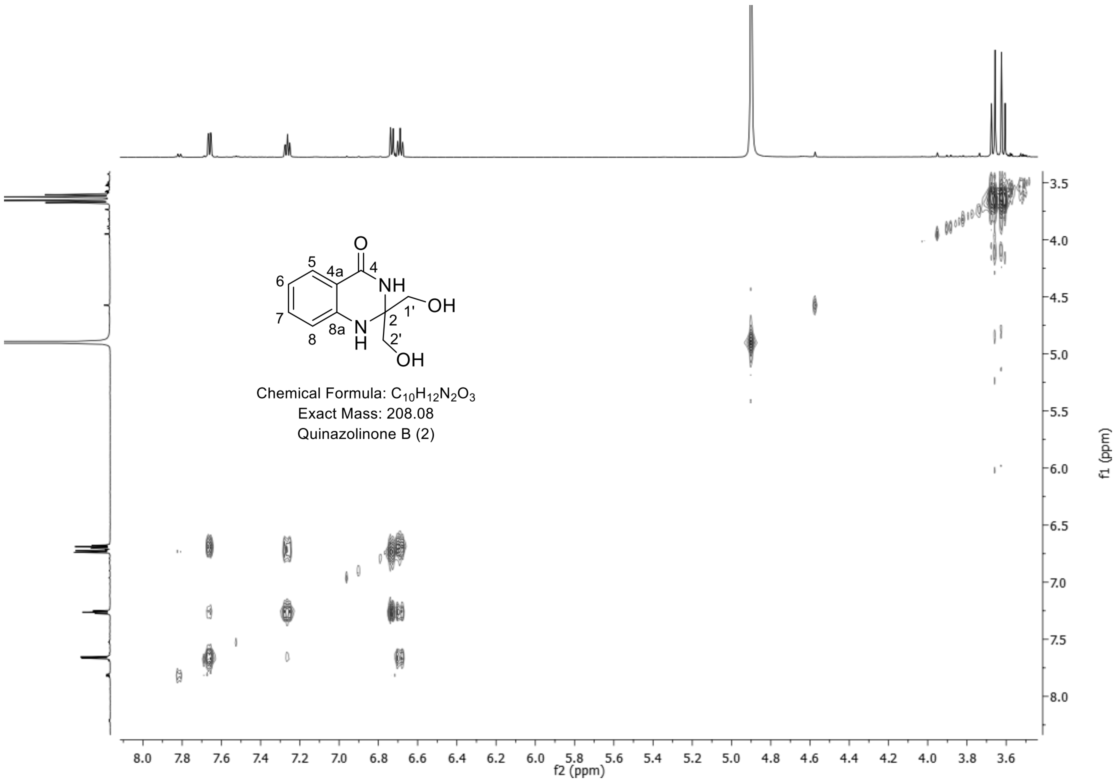

Fig. S14 ^{1}H-^{1}H COSY spectrum of *quinazolinone B* (**2**) in CD_3OD

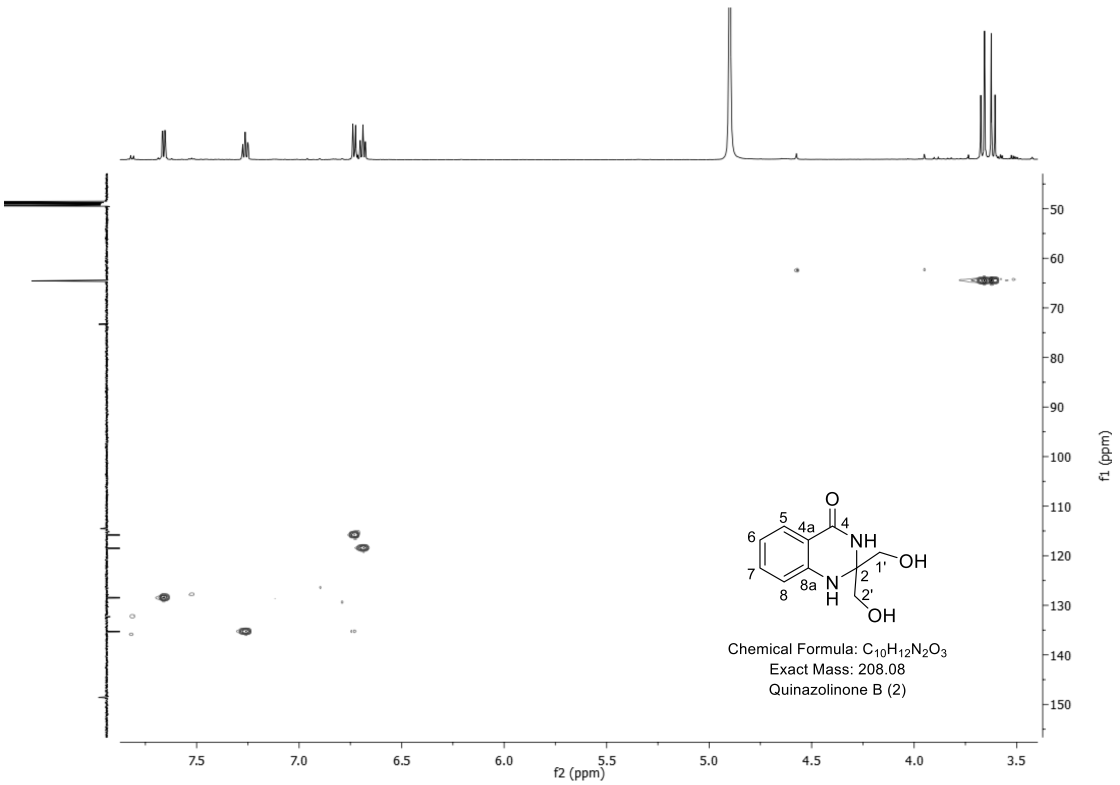

Fig. S15 HSQC spectrum of *quinazolinone B* (**2**) in CD_3OD

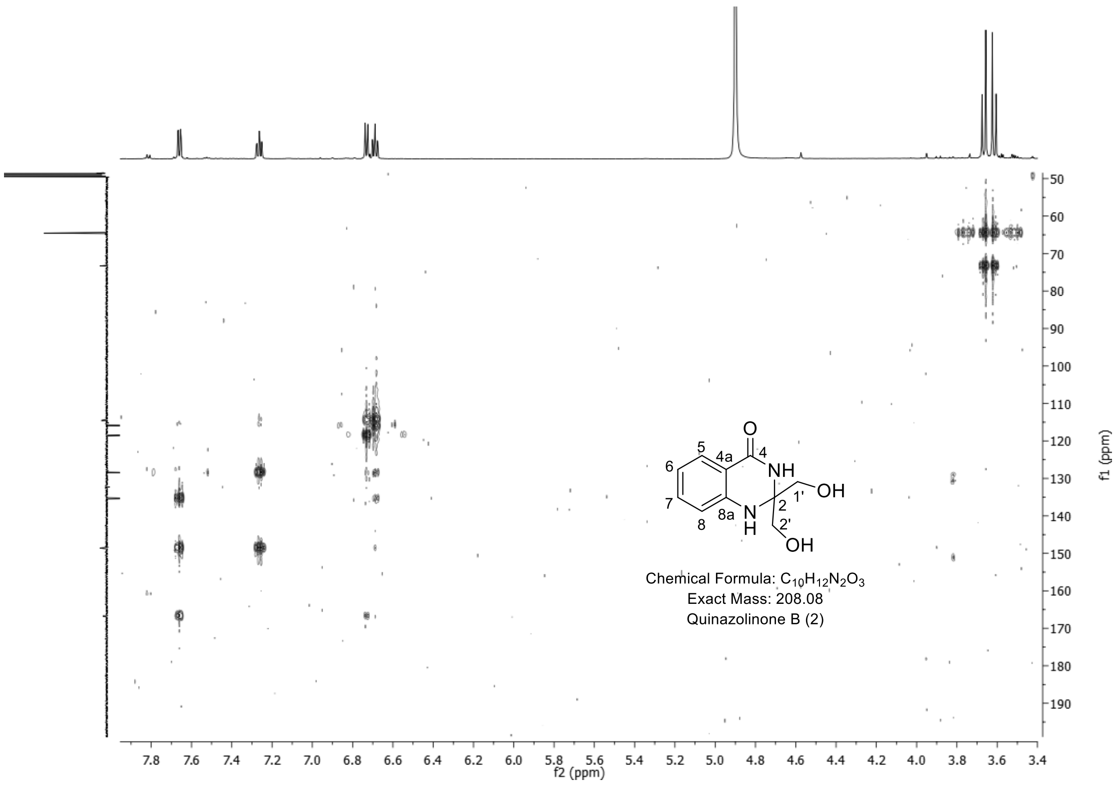

Fig. S16 HMBC spectrum of *quinazolinone B* (**2**) in CD_3OD

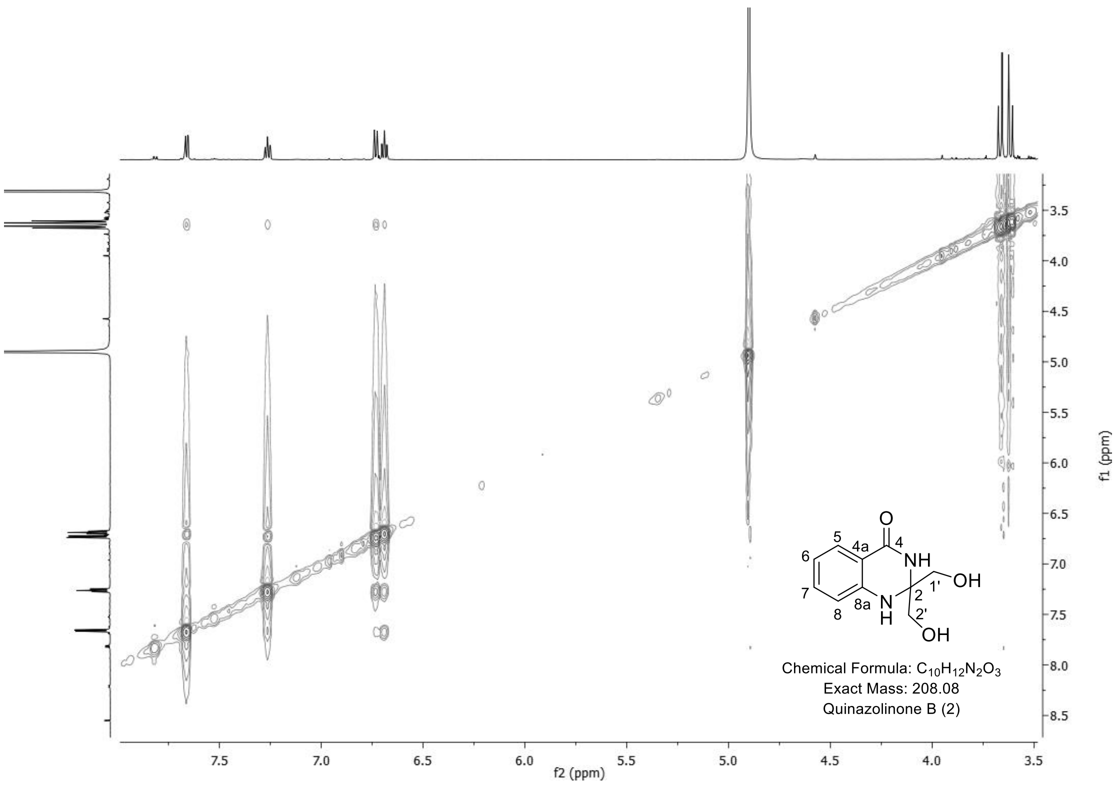

Fig. S17 ^{1}H-^{1}H NOSEY spectrum of *quinazolinone B* (**2**) in CD_3OD

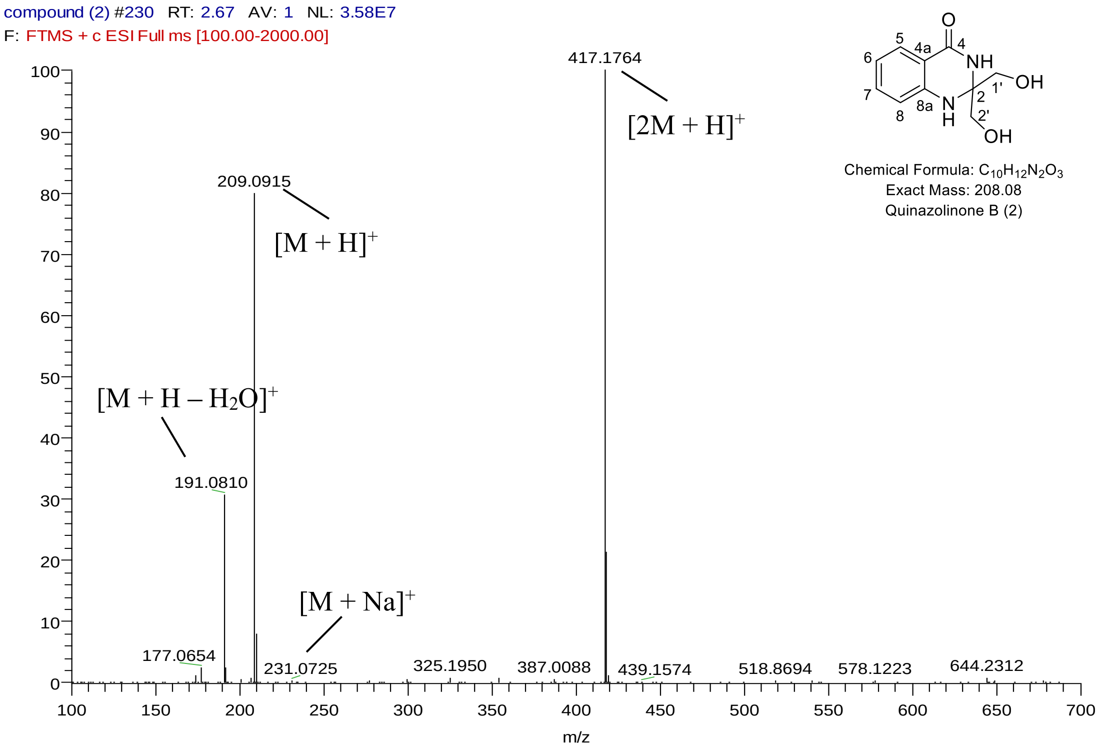

Fig. S18 HRMS spectrum of *quinazolinone B* (**2**)

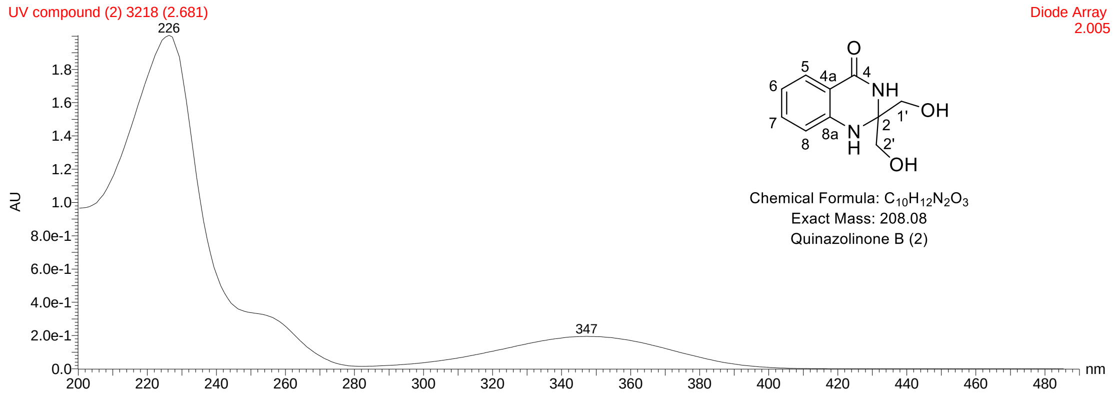

Fig. S19 UV spectrum of *quinazolinone B* (**2**)

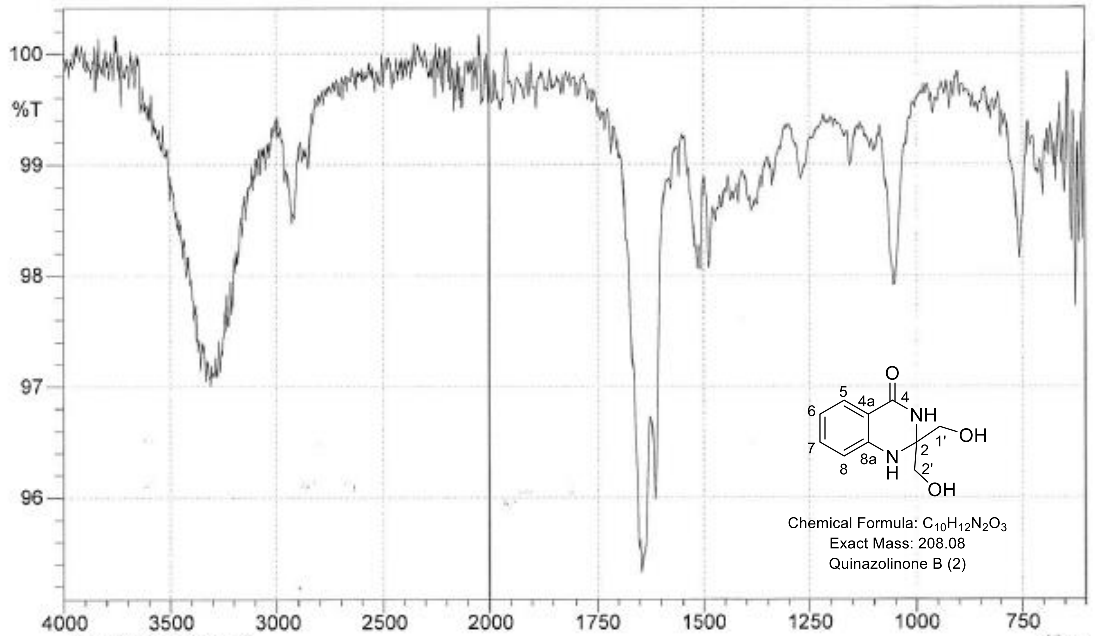

Fig. S20 IR spectrum of *quinazolinone B* (**2**)
